# Engineering and Optimization of Silicon–Iron–Manganese Nanoalloy Electrode for Enhanced Lithium-Ion Battery

**DOI:** 10.1007/s40820-017-0142-8

**Published:** 2017-03-17

**Authors:** Pankaj K. Alaboina, Jong-Soo Cho, Sung-Jin Cho

**Affiliations:** Joint School of Nanoscience and Nanoengineering, North Carolina Agricultural and Technical State University, Greensboro, NC 27401 USA

**Keywords:** Electrode engineering, Silicon nanoalloy, Calendering effect, Electrolyte wettability, High-density silicon anode

## Abstract

The electrochemical performance of a battery is considered to be primarily dependent on the electrode material. However, engineering and optimization of electrodes also play a crucial role, and the same electrode material can be designed to offer significantly improved batteries. In this work, Si–Fe–Mn nanomaterial alloy (Si/alloy) and graphite composite electrodes were densified at different calendering conditions of 3, 5, and 8 tons, and its influence on electrode porosity, electrolyte wettability, and long-term cycling was investigated. The active material loading was maintained very high (~2 mg cm^−2^) to implement electrode engineering close to commercial loading scales. The densification was optimized to balance between the electrode thickness and wettability to enable the best electrochemical properties of the Si/alloy anodes. In this case, engineering and optimizing the Si/alloy composite electrodes to 3 ton calendering (electrode densification from 0.39 to 0.48 g cm^−3^) showed enhanced cycling stability with a high capacity retention of ~100% over 100 cycles. 
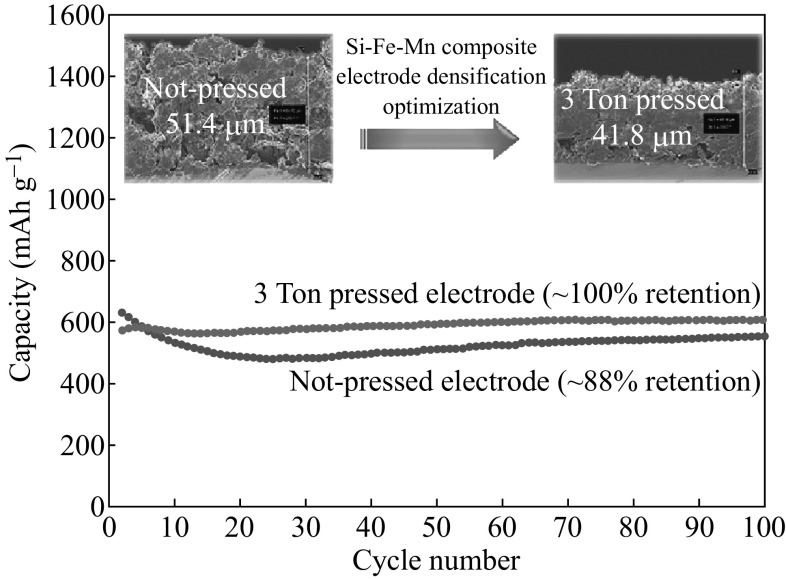

## Highlights

- Si–Fe–Mn alloy (Si/alloy) electrodes with high loading of 2 mg cm^−2^ were calendered at 3, 5, and 8 tons pressure and investigated on porosity, wettability, and electrochemical properties.
- Electrode engineering and wettability optimization balance are necessary to realize the true electrochemical potentials of the battery materials.

## Introduction

Batteries pour life power into the electronic devices, and there has been incredible development trying to stretch their energy densities, cycle life, and safety [1, 2]. Nickel–metal hydride batteries [3], nickel–cadmium batteries [4], and lithium-ion batteries (LIBs) [5] are some of the different popular batteries commercially available in the market. In industry scale, high emphasis is given on the electrodes calendering and wettability optimization by means of pressure injection of electrolytes for consistent and stable batteries. Rather, in the laboratory scale research, the progress in battery technology most times is primarily inclined toward the development of novel electrode active materials [6–8], followed by binders, electrolytes, and enhancement additives [9–13]. However, to realize the true potential of an electrode material, it requires different levels of engineering design and optimization which should also be focused [14, 15]. The same electrode material can be designed to achieve improved performances if parameters such as thickness, density, wettability, and porosity are controlled and optimized.

In previous works, researchers have reported the effect of calendering on electrode wettability and electrochemical properties. van Bommel et al. [16] showed the improvement in the LiFePO_4_ electrodes performance after calendering. Church et al. showed the changes in the pore structure and wettability after calendering of electrodes which can significantly impact the battery performance [17]. Kwade et al. discussed the impact of the compression by calendering on the interfacial structure, ion mechanics, and long-term electrochemical performance of LIBs [18]. Newman [19] presented a reaction zone model to optimize the porosity and thickness of electrodes knowing its importance. Similarly, various studies and mathematical models have been developed to understand the physical phenomena and compute the variations in electrochemical properties [20]. In this work, the fabricated silicon alloy electrodes were calendered at 3, 5, and 8 tons pressure. The densified electrodes were investigated in terms of porosity, electrolyte wettability, and long-term cycling to provide insight on silicon alloy electrode design optimization for improved electrochemical performance. The anode electrodes were made of silicon (Si) alloyed with iron (Fe) and manganese (Mn) as the active material. Si–Fe–Mn alloy material’s (hereafter denoted as Si/alloy) significance is its easy synthesis using a very low-cost and low-temperature process that includes only mechanical milling and drying. Moreover, Fe and Mn components in a silicon alloy anode improve the materials overall electrical conductivity [21] and are reported to be inactive with lithium (Li) alloying [22, 23] during lithiation–delithiation cycles, but their high ductile property acts as a buffer matrix for Li–Si alloy expansion and contraction—increasing the reversible capacity [24–26]. Furthermore, Mn combination can help to inoculate Fe, further improving the overall ductility of the metal alloy [27].The objective of this research is to engineer the high loading Si/alloy and graphite composite electrodes by investigating the properties variations with calendering at different pressures and optimizing to balance between the electrode thickness and porosity–wettability for improved electrochemical performance.

## Experimental

### Electrode Preparation and Calendering

Si/alloy was prepared by using a low-cost and low-temperature mechanical ball milling process. A commercial high-energy ball mill (ZOZ GmbH, Simoloyer) was used for the mechanical alloying with elemental powders of Si (99.99%), Fe (99.9%), and Mn (99.9%) in the stoichiometric of 80:18:2 wt%, respectively, as the starting materials. The powders were loaded into the machine’s hardened steel chamber with hardened steel balls of 4.7 mm diameter for milling. The milling cycle was characterized by a time interval of 45 s at 12 m s^−1^ (circumferential velocity), followed by 15 s at 8 m s^−1^ for 12 h and was fully carried out under super-high-purity argon atmosphere. The mass ratio of milling ball-to-powder materials was maintained as 20 to 1. The electrode slurry was prepared by first mixing 0.025 g of ketjen black (KB) and 1.35 g of polyamide-Imide (PAI) binder solution (14.8% binder and balance deionized water solution from Aekyung Chemical Co., LTD) in 2.6 g of deionized water for 4 h or more using magnetic stirrer at 100 RPM on a hot plate at 25 °C. KB was used as a conductive filler additive, and PAI binder was chosen because of their high carboxylic groups which are known to form strong bonds with metal surfaces and maintain good electrical contacts between the electrode particles [12, 28]. Active material was prepared by making a blend of the Si/alloy sample powder (40%) with MC20 (Mitsubishi Corp.—Synthetic Graphite) and SFG6 graphite (TIMCAL TIMREX^®^—Synthetic Graphite) in the ratio of 42 and 18%, respectively. Active material blend (0.945 g) was then mixed in the KB-PAI binder solution slurry using ACE NISSEI homogenizer at 5000 RPM for 15 min. MC20 (0.93 g) and SFG6 (0.4 g) artificial graphite were added in sequence to the slurry solution with each step mixed at 5000 RPM for 10 min. The final contents were mixed using a spatula, and again a final mixing was performed using the ACE NISSEI homogenizer at 5000 RPM for 15–30 min to get the homogenous electrode slurry. The order of materials for slurry making was decided based on the size and shape of the material components for homogeneous and good quality electrodes. The electrode preparation composition in total included Si/alloy (15.1 wt%), MC20 (53.1 wt%), SFG6 (22.8 wt%), PAI binder (8 wt%), and KB (1 wt%). The overall solid content used was around 40%. The resultant slurry was poured onto a Cu-foil, and doctor blade with thickness set to (0.15 ± 0.01 mm) was run at 1.2 speed unit of CV-400 Rotech Lab Coater to get a final active material loading of ~2 mg cm^−2^. The electrodes were put for drying in a vacuum oven at 110 °C for 10 h. The electrodes after drying were punched into 14-mm disks.

**Fig. 1:**
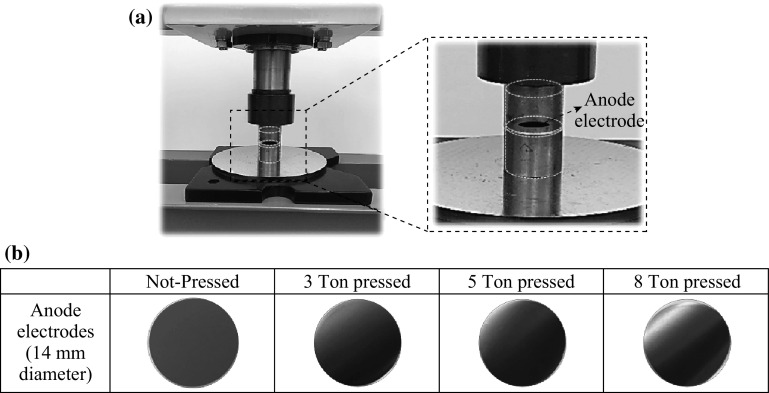
**a** Electrode calendering between the two steel bars using a bench press machine. **b** Optical images of the punched electrode disks of the not-pressed electrode and 3- , 5- , and 8-ton pressed electrodes

The punched electrodes were then calendered using a Dake Model value B-10 press and two 1” diameter × 1” length steel bars as shown in Fig. 1a. The electrodes were placed between the two steel bars and pressed at 3, 5, and 8 tons pressure and held for a minute before releasing the pressure. The steel bars make sure uniform load distribution, and the pressure was applied normal to the surface of the punched electrodes (14-mm disks, surface area 1.54 cm^2^). The electrodes after calendering were dried in vacuum oven at 110 °C for 2 h to remove any traces of moisture absorbed during the whole calendering process. After drying, the electrodes were transferred into an argon-filled glove box to build CR2032 coin cells for electrochemical testing.

### Electrodes Surface, Porosity, and Electrolyte Wettability Investigation

Calendering the electrodes decreases the surface area and porosity and affects the electrolyte wettability. To get a closer look on the surface of the calendered electrodes, Carl Zeiss Auriga-BU focused ion beam field emission scanning electron microscope was used for observations. In addition, the cross section of the electrodes was captured to see the thickness control with different calendering pressures used.

The influence of calendering on electrolyte wettability was interpreted using electrolyte–electrode contact angle measurements from Rame’-hart Contact Angle Goniometer using DROP Image version 2.6.1 software [29]. Ethylene carbonate (EC)/diethyl carbonate (DEC)/fluoroethylene carbonate (FEC)—5/70/25 (v/v) with 1 M LiPF_6_ from PanaxEtec Co., Ltd.-Starlyte was used as the wettability test electrolyte. The electrolyte (5 µL) was dropped on the electrodes using a fixed volume pipette, and the contact angles were measured just after dropping. The electrolyte (5 µL drop) quickly get absorbed by the electrodes, and as such, a slow-motion frame capturing the first electrode–electrolyte contact was used for the contact angle and wettability measurements.

Brunauer–Emmett–Teller (BET) surface area and porosity measurement analysis were performed using Micromeritics ASAP-2020 (Nitrogen sorption at 77 K). Two 14-mm electrode disks were used in the BET analysis. Nitrogen adsorption isotherms measurement was carried out in a relative pressure range from 0.04 to 0.25 and using the following degassing method. First, evacuation at 50.0 mmHg s^−1^ to 500 µmHg, and hold for 60 min. Second, temperature ramp at 10 °C min^−1^ to 100 °C, and hold at 100 °C for 120 min.

### Electrochemical Cell Assembly and Characterization

In the coin cells assembly, Li metal (15.6 mm diameter × 0.25 mm thickness) was used as a counter electrode with Celgard 2500 as the separator. 60 µL of 1 M LiPF_6_ in ethylene carbonate (EC)/diethyl carbonate (DEC)/fluorethylene carbonate (FEC)—5/70/25 (v/v) from PanaxEtec Co., Ltd.-Starlyte was used as electrolyte. During assembly, a total electrolyte wetting time of 30 min was allowed before crimping the cells in each case. After assembly, the coin cells get to rest in the glove box for 20 h before starting the electrochemical testing.

Electrochemical characterization was performed using a Toyo TOSCAT 3100 battery cycler. The cells were cycled at 0.1C rate for the formation cycle and then tested at 0.5C rate for 100 cycles in the potential range between 0.01 and 1.5 V. Electrochemical impedance spectroscopy (EIS) measurements were performed after the first cycle and after 100th cycle when the coin cell voltages reached 0.01 V (Lithiated state) using VMP3—Modular 16 Channels Potentiostat/Galvanostat/EIS machine (Scan frequency: 100 kHz–10 mHz; Amplitude: 10 mV).

## Results and Discussion

### Electrodes Surface, Porosity, and Wettability Characterization

**Fig. 2:**
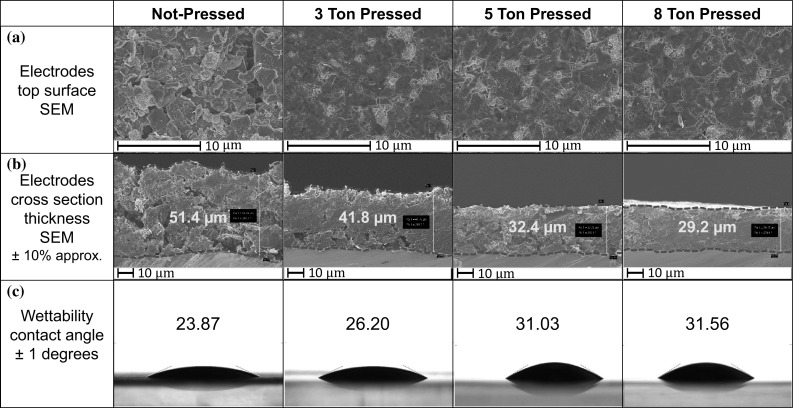
**a** SEM images of the top surface. **b** Cross-sectional SEM images and coating thickness measurements. **c** Electrode/electrolyte wettability contact angle measurement results of the not-pressed electrode and 3- , 5- , and 8-ton pressed electrodes

Figure 1b shows the optical images of the punched electrodes before and after calendering at 3, 5, and 8 tons. The increase in electrode shininess with pressing can be witnessed indicating the increase in smoothness upon electrode densification. SEM images in Fig. 2a row show that the not-pressed electrodes have a large number of porous structures on its surface, while after calendering the porous structures were pressed and pores get reduced making it a relatively smooth and uniform surface. Figure 2b shows the cross-sectional SEM images of the electrodes to measure the thickness variations with calendering, and also providing insight on the surface and core porous structures. The coating thickness is indicated using the dashed line. For 8-ton pressed electrode, Fig. 2b cross-sectional SEM, the electrode top surface was slightly visible during imaging which appeared as the bright white layer and was not considered in the cross-sectional thickness measurement as highlighted by the dashed lines. The not-pressed electrode has an average coating thickness of 51.4 µm which after calendering at 3, 5, and 8 tons compressed to 41.8, 32.4, and 29.2 µm, respectively, with an error bar of ±10% approx. for the thickness measurements. In addition, the cross-sectional SEM images in Fig. 2b row also show that not-pressed electrodes are more porous on the surface and in its core, while the calendered electrodes have a less porous structure with a very uniform surface. Calendering of electrodes increases the particle-to-particle contact and improves electrode thickness uniformity but on the other side severely reduces porosity and wettability calling for optimization requirement.

**Fig. 3:**
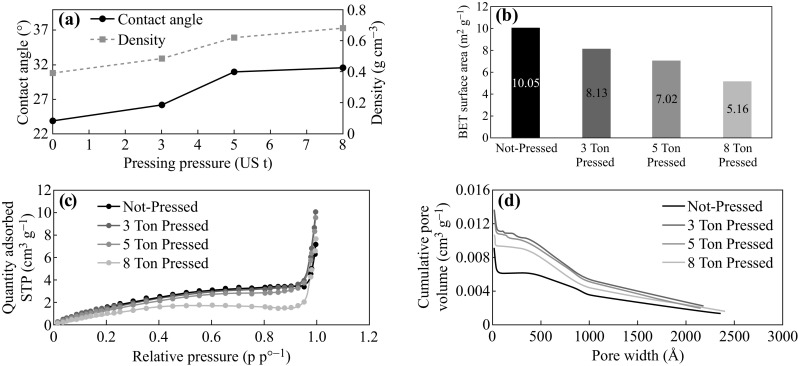
**a** Electrodes contact angle and density trend with applied calendering pressures. **b** BET-specific surface area results. **c** BET surface area nitrogen isotherms of the not-pressed and 3- , 5- , and 8-ton pressed electrodes. **d** BET porosity distribution curves—pore volume versus pore width of the same sample electrodes

Electrode calendering is required to increase the volumetric capacity of the electrode and to fabricate the electrodes with a uniform thickness of the coating. Nevertheless, calendering sizably reduced the surface and core porous structures of the electrodes which lead to poor electrolyte wettability. The electrolyte wettability investigations using the contact angle goniometer are shown in Fig. 2c, and the electrode details are summarized in Table 1 for the not-pressed and pressed electrodes. The electrode coating thickness measurements are taken from the SEM cross-sectional images in Fig. 2b. Similar electrodes with weights of 23.4 mg and active material loading of ~2 mg cm^−2^ were used for the wettability study. The active material density for the not-pressed electrode is calculated to be 0.39 g cm^−3^ and is densified to 0.48, 0.62, and 0.68 g cm^−3^ by calendering at 3, 5, and 8 tons, respectively. The contact angles measured for the electrode samples were found to increase with calendering indicating the relative decrease in porosity and electrolyte wettability [29]. The not-pressed electrodes showed a contact angle of 23.87° ± 1° which was less than the contact angles of the pressed electrodes due to its high porosity. As expected, the pressed electrodes showed higher contact angles of 26.20° ± 1°, 31.03° ± 1°, and 31.56° ± 1° for the 3- , 5- , and 8-ton pressed electrodes, respectively (shown in Fig. 2c), indicating reduced wettability property. Figure 3a illustrates the trend in the electrode density and wettability contact angles with calendering. The differences in the contact angles of the not-pressed and pressed electrodes show the decrease in wettability of the electrodes with calendering. The decreased wettability leads to incomplete electrolyte filling and in addition demands for increased electrolyte wetting time. From the contact angle wettability investigations (Fig. 2c), the densified electrodes with 3 ton pressing showed considerably good wettability compared to the not-pressed electrodes. On the other hand, the electrodes with 5- and 8-ton pressed electrodes have much higher densities but stress for more extended wetting time requirements.

**Table 1:** Electrode thickness, weight, active material loading, density, and electrolyte wettability contact angle results for not-pressed and 3- , 5- , and 8-ton pressed electrodes

	Not-pressed	3 ton pressed	5 ton pressed	8 ton pressed
Electrode coating thickness (µm) ±10% approx.	51.4	41.8	32.4	29.2
Electrode weight (mg)	23.4	23.4	23.4	23.4
Material loading (mg cm^−2^)	2.00	2.00	2.00	2.00
Active material density (g cm^−3^) ±10% approx.	0.39	0.48	0.62	0.68
Wettability contact angle (±1°)	23.87	26.20	31.03	31.56

BET surface area and porosity measurements were used to confirm the variations in the active surface area and porous structures with calendering of electrodes. The nitrogen gas adsorption isotherms are shown in Fig. 3c. The not-pressed electrodes have higher porosity and higher surface area which can adsorb more quantity of nitrogen gas, and hence, the isotherm adsorption level is greater for not-pressed electrodes. Likewise, the calendered electrodes showed reduced porosity and reduced surface area. The calendered electrodes showed relatively less amount of surface gas adsorption, and the adsorption isotherm curves were falling below the not-pressed electrode as shown in Fig. 3c. The results indicate that the not-pressed electrodes have a higher surface area of 10.05 ± 1 m^2^ g^−1^. Pressed electrodes showed comparatively less surface area of 8.13 ± 1, 7.02 ± 1, and 5.16 ± 1 m^2^ g^−1^ for 3- , 5- , and 8-ton pressed electrodes, respectively, due to the decrease in surface porosity with calendering which is agreeing with the SEM images and the contact angle results in Fig. 2. The pore size distribution comparison is shown in Fig. 3d. The not-pressed electrodes have an average adsorption pore width of 43.9 nm, while the calendered electrodes showed 76.4, 84.3, and 91.6 nm for 3- , 5- , and 8-ton pressed electrode, respectively (±10 nm approx.). From the BET results, the single point adsorption total pore volume of pores less than around 500 nm was found to be 0.011 cm^3^ g^−1^ for the not-pressed electrodes and little higher around 0.014 cm^3^ g^−1^ (approx.) for the calendered electrodes, respectively. The small pore width and smaller pore volume for the not-pressed electrodes again indicate that they have a large number of smaller pores and higher surface area compared to the pressed electrodes. However, the pressed electrodes were found to have relatively higher pore sizes and pore volumes compared to the not-pressed electrodes as shown in Fig. 3d. The results indicate the closing of a large number of smaller pores with calendering. The smaller pores are compressed and joined to form bigger structures showing good agreement with the decreasing trend in the surface area of the electrodes with increasing calendering pressure (Fig. 3b).

### Electrochemical Performance

Densification of the electrodes showed stable cycle performance compared to the not-pressed electrodes. The coin cells made were galvanostatically charged–discharged at 0.1C rate in the first formation cycle and tested at 0.5C for the next 100 cycles. The electrode loading for all the samples was high at around 2 mg cm^−2^.

**Fig. 4:**
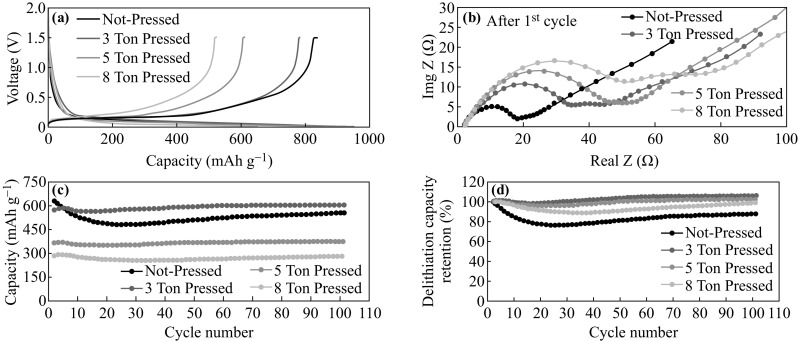
**a** Formation cycle at 0.1C rate of not-pressed, 3-ton pressed, 5-ton pressed, and 8-ton pressed electrodes. **b** Electrochemical impedance measurements of the same samples after the first cycle at 0.5C rate. **c** Delithiation cycling capacity of the same samples over 100 cycles at 0.5C rate. **d** Delithiation capacity retention of the same samples over 100 cycles at 0.5C (potential window = 0.01–1.5 V, active material loading ~2 mg cm^−2^, and 1C = 620 mA g^−1^)

In Fig. 4a, not-pressed electrode cell exhibited the highest delithiation charge capacity of 837.5 mAh g^−1^ with an initial Coulombic efficiency of 88.1%. The calendered electrode cells 3, 5, and 8 tons showed a reversible capacity of 782.7, 609.9, and 522.2 mAh g^−1^ in the formation cycle with a Coulombic efficiency of 86.4, 84.3, and 80.5%, respectively. The decrease in charge capacity and Coulombic efficiency with increasing calendering is recognized to the decrease in surface and core active pores of the electrodes due to densification. Moreover, the electrochemical activity was also limited by the poor electrolyte wettability arising due to the reduced pores in the calendered electrodes. Similarly, the EIS measurements after the first cycle at 0.5C (Fig. 4b) show that the electrode impedance increases with increasing densification from not-pressed electrodes to 3- , 5- , and 8-ton pressed electrodes. Electrodes densification improves the particle-to-particle contact with pressing. However, the electrochemical performance is affected due to reduced electrolyte wettability and reduced electrochemical activity indicating the need for densification control and wettability optimization. The pressed electrodes with incomplete electrolyte fillings or poor wettability demand for increased electrolyte wetting time which again requires optimization. In this case, an overall waiting time of 30 min was applied for electrolyte wetting for all the pressed and the not-pressed electrodes during cell assembly.

The EIS and cycling capacity indicate that the 5- and 8-ton pressed electrodes with the poorest wettability would require much longer wetting time for reaching their true electrochemical potential capacity. In general, longer wetting time requirements would be stressful for the fast productivity of batteries and can only be considered to some degree upon optimization and balancing to desired electrochemical properties. Figure 4c demonstrates the stable cycle behavior of calendered electrodes compared to the not-pressed electrodes, but with a capacity tradeoff which was considerable of 3- and 5-ton pressed electrode cells. In Fig. 4c, the not-pressed electrode cell showed a delithiation charge capacity of 629.3 mAh g^−1^ in the first cycle which faded to 552.6 mAh g^−1^ at 100th cycle at 0.5C cycling. The not-pressed Si/alloy electrodes showed high capacity at the first cycle and started to fade gradually for the next 20 cycles before stabilizing. The gradual fading for the initial cycles in not-pressed Si/alloy electrode cell is attributed to its relatively high lithium loss in solid electrolyte interphase (SEI) formations due to its high active surface area (Fig. 3b). It was also observed that during the cycle stabilizing stage, the capacity gradually increases for next following cycles which can be attributed to the increased lithiation into new active sites of the electrode as electrolyte wettability (i.e., electrolyte activating new open sites of the electrodes and also impregnating into deeper electrode core) gets improved with extended cycling. Similar cycling behavior was reported in the prior Si anode works [30, 31]. The calendered electrode cells showed a first cycle capacity of 574.2, 368.0, and 288.3 mAh g^−1^ for the 3- , 5- , and 8-ton pressed electrodes, respectively. The capacity of the calendered electrode cells was lower because of the reduced active surface area (low surface area for pressed electrodes, Fig. 3b) and poor electrolyte wettability (increasing contact angle with pressing, Fig. 2c) limiting the overall electrochemical activity. However, the calendered electrode cells showed very stable cycle retention behavior compared to the not-pressed electrode cell due to improved particle-to-particle contact with pressing and improved particles connectivity. The capacity after 100 cycles for the 3- , 5- , and 8-ton pressed electrode cells was 606.1, 375.7, and 284.7 mAh g^−1^, respectively.

**Fig. 5:**
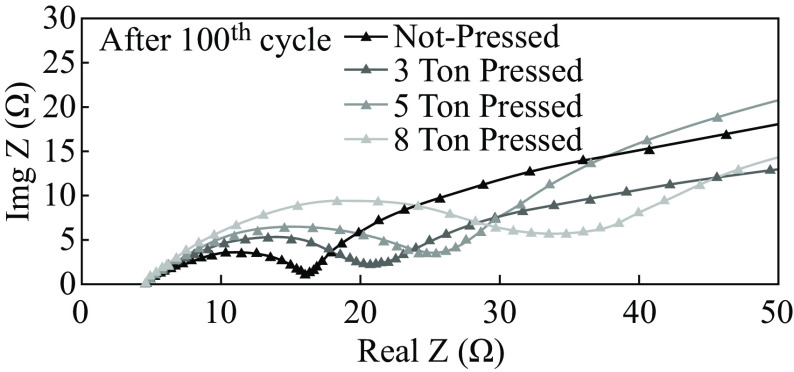
Electrochemical impedance measurements of the not-pressed, 3-ton pressed, 5-ton pressed, and 8-ton pressed electrodes after 100 cycles at 0.5C rate

For a better picturing of the stable electrochemical performance achieved with calendering, the delithiation capacity retention of the samples over 100 cycles (at 0.5C) is plotted in Fig. 4d. The not-pressed electrode cells showed a capacity retention of 87.8%, while the pressed electrodes showed capacity retentions almost close to 100% as shown in Fig. 4d. EIS was performed again, now after 100 cycles, Fig. 5, which showed a similar trend in activity as observed in the first cycle EIS results (Fig. 4b).

It should be noted that the same Si/alloy electrode material showed very different electrochemical behavior with different calendering. All the calendered electrode cells showed stable cycling behavior compared to the not-pressed electrode cell, but the overall capacity was lower with increasing calendering pressure due to reduced electrolyte wettability. Although the capacities are lowered, the stable cycle behavior with calendering would also make it easier to design high-density full cell batteries. In this case, 3-ton pressed Si/alloy electrode cell with a considerable capacity tradeoff showed high density and excellent cycle stability compared to the not-pressed Si/alloy electrode cell. Electrodes densification improves the particle-to-particle connections with pressing. However, the electrochemical performance is affected due to reduced electrolyte wettability and reduced electrochemical activity indicating the need for densification control and wettability optimization. Si/alloy electrodes with 5 and 8 tons pressed also showed stable cycling, but the capacities are very low to be considered. Therefore, optimization and balancing between the calendering pressure and electrolyte wettability property can help tune to the best electrochemical properties of the battery materials.

## Conclusions

Si–Fe–Mn alloy electrodes with high loading of 2 mg cm^−2^ were densified and investigated on porosity, wettability, and electrochemical properties for optimized performance compared to the not-pressed electrodes. Calendering is found to be influencing the long-term cycling and can be made beneficial with densification pressure and wettability optimization for the same electrode material (in this case 3-ton pressed electrodes). As a scientific rule of thumb, it should be considered that batteries are not always about just the right materials, but engineering and optimization can bring about a significant difference in the electrochemical properties. In short, electrode densification and wettability optimization balance are necessary to realize the true electrochemical potentials of the materials for batteries.
